# The use of a tablet-based app for investigating the influence of autistic and ADHD traits on performance in a complex drawing task

**DOI:** 10.3758/s13428-021-01746-8

**Published:** 2022-01-11

**Authors:** S. Savickaite, C. Morrison, E. Lux, J. Delafield-Butt, D. R. Simmons

**Affiliations:** 1 School of Psychology and Neuroscience, University of Glasgow, Room 531, 62 Hillhead street, Glasgow, G12 8QB Scotland; 2 Laboratory for Innovation in Autism, University of Strathclyde, Glasgow, UK

**Keywords:** Autism, ADHD, iPad app, Complex drawing task

## Abstract

This paper describes a smart tablet-based drawing app to digitally record participants’ engagement with the Rey-Osterrieth complex figure (ROCF) task, a well-characterised perceptual memory task that assesses local and global memory. Digitisation of the tasks allows for improved ecological validity, especially in children attracted to tablet devices. Further, digital translation of the tasks affords new measures, including accuracy and computation of the fine motor control kinematics employed to carry out the drawing Here, we report a feasibility study to test the relationship between two neurodevelopmental conditions: autism spectrum disorder (ASD) and attention-deficit/hyperactivity disorder (ADHD). The smart tablet app was employed with 39 adult participants (18-35) characterised for autistic and ADHD traits, and scored using the ROCF perceptual and organisational scoring systems. Trait scores and conditions were predictor variables in linear regression models. Positive correlations were found between the attention-to-detail, attention-switching and communication subscales of the autistic trait questionnaire and organisational scores on the ROCF task. These findings suggest that autistic traits might be linked to differential performance on the ROCF task. Novelty and future applications of the app are discussed.

## Introduction

Multisensory information from the world around us needs to be integrated, processed, organised and understood. Our ability to find patterns in the surroundings is often called “perceptual organisation” (Wagemans et al., 2012). Based on task demands, we use different types of perceptual organisation. The ability to extract the “big picture” is called global processing, and the ability to notice the details is called local processing (Simmons & Todorova, 2018). We often use these two types of perceptual organisation interchangeably. However, distinct preferences have been reported in some populations (e.g. autism: Kandaloft, Didehbani & Krawczyk, 2019; ADHD: Wang & Reid, 2011).

Autism spectrum disorder (ASD)^1^ is defined in the Diagnostic and Statistical Manual of Mental Disorders, Fifth Edition (DSM-5) as a condition characterised by difficulties with social communication, sensory processing and repetitive behaviours (American Psychiatric Association [APA],  2015). Motor issues associated with autism are becoming increasingly recognised and are considered by some to be a core aspect of the condition (Fournier et al., 2010; Trevarthen & Delafield-Butt, 2013). A recent meta-analysis by NHS Digital (2016) suggests that over 1% of the UK population is diagnosed with ASD. The latest study by the Centers for Disease Control and Prevention in the United States suggests a prevalence of close to 2% there (Baio et al., 2018). Distinct cognitive and perceptual styles have been observed in autism (Baron-Cohen, 2004; Simmons et al., 2009), but there is, as yet, no theoretical consensus on the underlying causes.

An early attempt at explaining the apparently distinctive visual processing style in autism was the weak central coherence (WCC) theory (Frith, 1989). The empirical basis of this theory was atypical perceptual organisation demonstrated in some visuospatial tasks, such as the embedded figures task and the block design task, and the differential impact of visual illusions (Happé, 1996). WCC suggests that autistic individuals outperform controls in these tasks due to “better attention to, and memory for, local details, but lessened global processing” (Fletcher-Watson & Happé, 2019). Enhanced perceptual functioning (EPF) theory (Mottron, 2001; Mottron et al., 2006) offers a subtly different explanation, in terms of enhanced local processing in autism without the obligatory (even if detrimental to task performance) global precedence found in non-autistic individuals. Happé and Frith’s (2006) revision of WCC (“weak coherence”) proposed superior local processing as a default “cognitive style” which could be overridden by explicit instructions (see also Koldewyn et al., 2011). Empirical support for any form of local versus global processing differences between autistics and non-autistics has, however, been patchy, with one thorough meta-analysis suggesting that, at least in static patterns, the only robust effect was the disruption of speed of global performance by local noise (Van der Hallen et al., 2015). Even this result has been called into question (Chamberlain et al., 2017). Empirical research which has employed sizable samples and extensive test batteries paints a complex picture, with task performance varying with IQ, gender and age, as well as diagnostic status (Van Eylen et al., 2018). More recently, there is a suggestion that disruption of perceptuo-motor coherence in the autism spectrum may be due, in part, to brainstem neuroanatomical and functional differences, especially noted in its subtle, but significant sensorimotor control differences (Bosco et al., 2019; Dadalko & Travers, 2018; Delafield-Butt et al., 2021).

Further accounts also discuss the hypothesis that the processing method used by an autistic individual is more driven by the attentional demands of the task (Plaisted et al., 1999). For example, enhanced perceptual load (EPL) theory (Remington et al., 2012) posits that autistic individuals inherently possess enhanced perceptual abilities. Therefore, perceptually complex tasks are less demanding, and the remaining capacity can allow for more distractor processing. Dual-coding theory (Smith & Milne, 2017), on the other hand, builds on Glyn Humphrey’s model of object processing and describes two parallel routes for encoding spatial features. However, local and global processing in autism is still a highly debated topic without a clear consensus (see Simmons & Todorova, 2018, for an overview). Moreover, similar debates around visual processing have extended to other neurodevelopmental conditions. For example, different performance on visuospatial tasks has been linked to both autism and attention-deficit/hyperactivity disorder in diagnosed groups (Wang et al., 2018).

Attention-deficit/hyperactivity disorder (ADHD) is defined in the DSM-5 as a neurodevelopmental disorder exhibiting patterns of inattention, hyperactivity and impulsivity (APA, 2013). Ebejer et al. (2012) estimated that 3–5% of the adult population in the UK is affected by ADHD. Autism and ADHD often co-occur, with an estimated 30–50% of autistic adults meeting ADHD diagnostic criteria (Rau et al., 2020). Autism and ADHD are distinct but related conditions, and when they co-occur, they often interact (Taurines et al., 2012). According to the findings of Song and Hakoda (2015), children with ADHD demonstrated a reduced preference towards global processing, contradicting DSM-5 (APA, 2015) criteria where failure to pay close attention to detail was emphasised. This finding was further supported by Cohen and Kalanthroff (2019) and Kalanthroff et al. (2013), suggesting that individuals with ADHD may experience a local processing bias. Local and global processing styles are not as extensively researched as in autism, and therefore, no formulated theory is available. However, recent findings suggest that autism and ADHD may share similarities in their overall visual processing style that may explain the overlap of symptoms (e.g. behavioural issues, difficulties in social situations). Currently, the extent of this overlap remains unclear (Groom et al., 2017) and warrants further investigation.

**Fig. 1:**
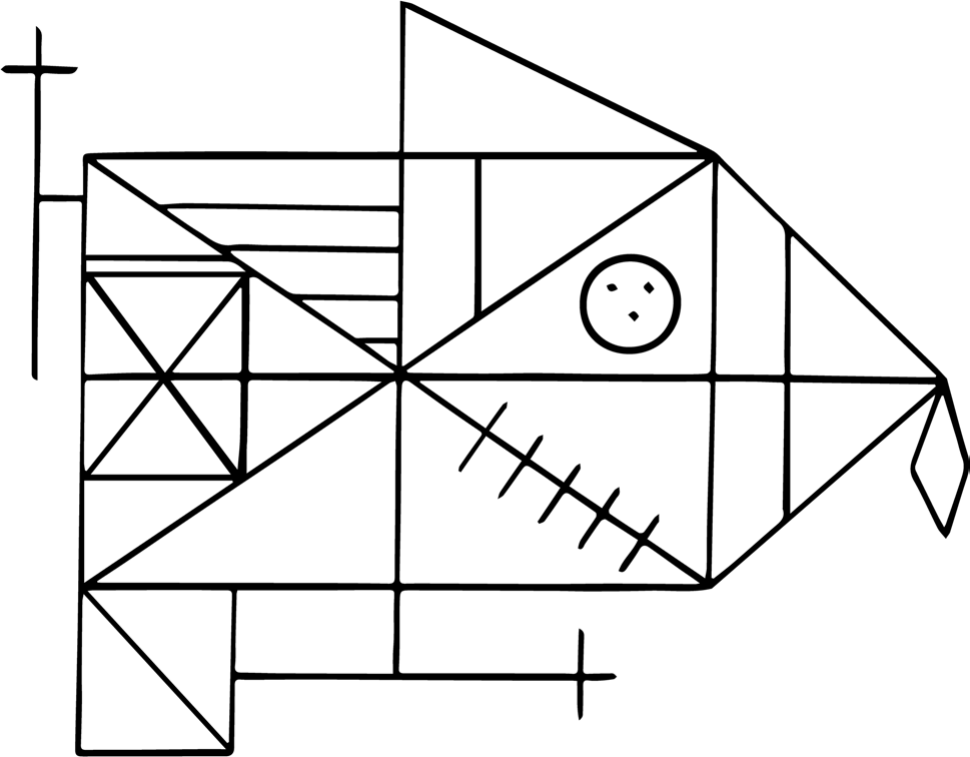
The Rey Osterrieth Complex Figure

Many different tasks have been used to investigate local-global visual processing differences in Autism and ADHD, but one of the most popular has been the Rey-Osterrieth complex figure (ROCF). This task is an established test for visuospatial memory, sensory processing style and executive function (Molitor et al., 2018; Shin et al., 2006; Watanabe et al., 2005). The ROCF was designed in the 1940s (Osterrieth, 1944; Rey, 1941) and was originally used for neuropsychological assessment (Fig. 1). Participants are first asked to copy the figure. Then the figure is taken away and participants are asked to draw it again from memory. Often a third recall condition is used where participants are asked to draw the figure again after a short delay. The ROCF has been used previously in local and global processing assessment in autistic and ADHD populations (Catanzaro, 2005; Kuschner et al., 2009; Minshew & Goldstein, 2001; Schlooz et al., 2006; Seidman et al., 1995;Tsatsanis et al., 2011 ; Van Eylen et al., 2018).

Developmental studies of the ROCF are of particular interest to the current work. Akshoomoff and Stiles (1995a, 1995b) provided valuable insight into developmental changes in ROCF tasks. They found that, before the age of 9 years, neurotypical children struggled to appreciate the figure as a coherent whole, instead adopting a piecemeal approach. This is similar to the method of processing proposed in WCC theory, where global elements of ROCF, such as the large rectangle and intersecting lines are drawn first and details (small lines, diamond and the “little face”) filled in later for “globally” inclined participants. The opposite would be expected in autistic and ADHD groups (Tsatsanis et al., 2011; Van Eylen et al., 2018). The ROCF has also been used as a measure of executive function in studies with individuals diagnosed with both ADHD and ASD, focusing on the organisational approach to the task. Akshoomoff and Stiles (1995b) found that the strategy adopted in the copy condition influences the manner in which children will draw it from memory, and therefore their overall performance.

The ROCF task is a widely used neuropsychological tool and the majority of literature on various scoring systems comes from clinical populations (e.g. epilepsy, individuals with brain lesions; Shin et al., 2006). In research into local and global processing in autism and ADHD, the most useful aspect of scoring is the ability to measure the features, primarily focusing on accuracy. Inclusion or absence of details may provide evidence for enhanced local processing and/or fragmented perception, often reported in first-hand accounts by autistics. Therefore, the perceptual and organisational scoring systems are the most suited for our study.

The nuances of how visual processing styles vary in neurodivergent individuals are still not fully understood. Different scoring systems for the ROCF task are an attempt to measure these nuances by altering which elements of the figure are considered most significant. However, in order to obtain a more complete account of performance in this task we should also measure temporal and kinematic aspects.

## Technological advances and their adaptation in research

Mobile technology provides various useful features, including flexible multimedia content and storage, portability and affordability (Vlachou & Drigas, 2017). Moreover, technological developments have integrated several movement and touch sensors into mobile devices such as phones and tablets, allowing unprecedented access to data on interaction accuracy and reliability (Millar, 2012). Mobile tools in neurodevelopmental research have been predominantly used for assessment and intervention, whereas computer-assisted learning (CAL) has been explored only recently (Fletcher-Watson, 2014). Technology-based approaches have been adapted for teaching literacy, emotion regulation and social skills. Research participants respond positively to technology and are often more verbal and interactive with touchscreen equipment (Fletcher-Watson et al., 2016).

Household tablet use increased from 8% in 2011 to 78% in 2017 (Kirkorian et al., 2020). Several studies have explored this rapid transition, predominantly in children, as it involves motor and cognitive elements. Growing access to touchscreen technology is one of the contextual factors contributing to development. Touchscreen technology is on track to replace the traditional forms of writing and drawing, and might have cascading effects on fine motor skills.

Kagohara et al. (2012) assessed the viability of iPads and similar technology for individuals with developmental disabilities. The majority of the 15 studies reviewed found positive results. Moreover, tablets have been successfully used in autism research (Vlachou & Drigas, 2017; Loth & Evans, 2019) for assessment, intervention and entertainment. Communication apps such as Speak4Yourself give a voice to non-verbal autistic individuals. Bishop (2003) reported on the PARLE app, which translates confusing language, such as metaphors, into easier meanings for autistic individuals to understand. Digital, specifically tablet, interventions have been found to raise social acceptance of augmented or alternative communication methods (Vlachou & Drigas, 2017).

Similarly, Anzulewicz et al. (2016) successfully developed a novel, smart tablet game for early identification of autism in young children by collecting tablet sensor data (inertial and touch screen sensors). This particular app employs a serious game approach with machine learning for identification of preschool children with ASD useful in screening and diagnostic services, and is currently under Phase 3 multisite diagnostic trial (Millar et al., 2019). Children with motor challenges have been shown to need additional time to learn how to navigate the device, but engagement ratings made up for this downside. Tablet-based intervention in autism has been claimed to result in communication improvement, successful learning of basic concepts, more self-talk, monitoring and recording of real-time data (Chmiliar, 2017). Tablet-based serious games have also been investigated in other contexts, such as sensory processing differences (Zakari et al., 2017). In short, tablet-based drawing provides more useful data and, due to its familiarity and ease of use, is likely to be more engaging for participants than traditional pen-and-paper-based approaches.

## ROCF task for tablets

The quickly developing world of technology has significantly improved touch screen and pen tablet response time for handwriting and drawing tasks. The new Galaxy Note 20 has a 120 Hz refresh rate and a 9 ms response time (Samsung, 2020). The iPad was ranked as one of the most responsive touchscreen tablets in 2013 (Agawi, 2013). Although not the most advanced tablet in the market today, with the addition of the Apple Pencil (Apple, 2015), it is still one of the top devices used for handwriting and basic drawing tasks.

Improvement in the screen response time inevitably led to an adaption of classical “pen and paper” tests, such as the ROCF, for tablet technology. Riordan, Lombardo and Schulenberg (Riordan et al., 2013) found mixed results on how much participants preferred interacting with the tablet compared to the standard pen and paper ROCF task. Engagement with a novel tool, i.e. tablet, was rated favourably; however, participants preferred pen and pencil for the task itself. This study was published at a time when tablet functionality was not as advanced as it is now because the touch screen response and refresh rates were poor. The ROCF task was used again in 2018 to investigate executive function in ADHD. No differences from the pen and paper test were reported (Hyun et al., 2018). The task itself was the same as the standard version. The images were further analysed using Gaussian filters in MATLAB. The number of pixels was calculated and compared to the reference image of the ROCF. Although the automation of image extraction and processing is a good idea, it does not add any further information on performance in the task.

This paper will introduce an iPad-based app called *LetsDraw,* which enables free drawing on a blank canvas, and coordinates with timestamps can be exported to recreate the real-time interaction. The temporal dimension introduced in this experiment is a more efficient way to identify the order in which the elements of ROCF are drawn. Standard pen-and-paper tests often use different coloured pens to identify time increments, which is a cumbersome and crude method. Although additional measures of pressure and kinetics can be included in the data collection, for the purposes of this study we are introducing the temporal element only. We wish to test the feasibility of the tool before building on additional measures of performance.

## Feasibility study

The aim of the feasibility study is to explore the relationship between autistic and ADHD traits, and ROCF scores using a tablet-based app. We expect decreased accuracy in delayed conditions; however, the effects of autistic and ADHD traits on the overall performance are difficult to predict, due to mixed results in current literature. Organisational scores, measuring how well participants comply with the global processing of the ROCF, are expected to decrease with higher scores on autism and ADHD questionnaires.

The app and the feasibility pilot study description will illustrate how the app can be used in research. The standard Rey-Osterrieth complex figure task will assess local and global processing in participants differing in autistic and ADHD traits. Although tablet devices have been used previously for the task in autism and ADHD (Canham et al., 2000; Hyun et al., 2018), none of them have yet combined spatial and temporal drawing measurements. Therefore, we cannot predict how temporal measures will be different between our participant groups. making this an exploratory study.

## Methods

### Participants

Data were collected between January and March 2019 at the School of Psychology, University of Glasgow. Participants were recruited via an online subject pool. Three out of the original 42 participants were excluded from the final analysis due to missing data. The mean age of participants was 21.8 (SD = 2.4) years. Twenty-nine identified as female, eight as male and two as other; 69.2% of participants were native English speakers, and three out of 39 were left-handed. Most of the participants were undergraduate students at the University of Glasgow. Although age and educational background cannot be generalised to a wider population, our sample provides some cultural diversity, as not all were native English speakers. However, extra care must be taken when generalising data collected from such a sample, no matter how diverse. As this was a feasibility study we are accepting the limitations of our sample size and limited generalisability of the findings.

## Materials

### Apparatus

The task was completed using the *LetsDraw* app as described in the app description portion of this paper. An iPad Mini 2 (Retina/2nd Gen) 1.3 GHz Apple A7 1 GB model A1489 (EMC 2695*) was used for this experiment. Participants used their touch screens to draw the figure (i.e. using the finger and not the stylus). The size of the tablet was 200 × 134.7 ×7.5 mm and it was 331 g in weight. The second-generation iPad mini has a 7.9-inch (diagonally) LED backlit multi-touch display with IPS technology and a resolution of 326 pixels per inch. The screen refresh rate is 60 Hz.

### *LetsDraw* app

The *LetsDraw* app is an extension of *studyDraw* (Lux, 2016) developed by Erin Lux at the University of Strathclyde using the Swift programming language via XCode (2003)^2^.

Basic XCode knowledge will be sufficient to manipulate and run the app together with the detailed description provided in the supplementary materials at https://sites.google.com/view/letsdrawapp.

Apple continuously updates their software, and the iOS must be up to date on the *home* device (i.e. laptop/desktop computer) and the *test* device (i.e. iPad/iPhone)^3^. In addition, XCode has recently been updated to the XCode Beta (2020) version, which has slightly modified the initial code of the app. The original code has now been debugged and updated for future use. However, the functionality described in this paper has not been altered by this update.

The t*est* device used for this paper was an iPad mini 2 (Retina/2nd Gen) 1.3 GHz Apple A7 1 GB model A1489 (EMC 2695*). Once the build has been successful the app will be automatically installed onto the *test* device.

When opened, the app will display a blank screen with a New option in the middle. Pressing this option will start the session. The next screen will ask the user to name the session. This name will later be extracted with the rest of the data from the session. The next screen will have two options: Start and Restart. Restart allows the user to go back to the beginning in case of an error.

Finally, a blank canvas will be presented where the drawing can be completed. This screen will be displayed for the length of time preselected in the code. When the session is finished the original screen with the New option will appear again. This will allow the next run. The app can be terminated by pressing the “Home” button on the device.

Multiple sessions can be run on the *test* device and each one will be recorded and stored. In order to extract the data, the *test* device will have to be connected to the *home* device. Each file is saved as a .csv^4^ file, which includes x and y coordinates and timestamps. Time elements are recorded every 16 ms, which results in approximately 1500 data points. This allows for the most accurate representation of the figure drawn; however, future iterations of the experiment could investigate the minimum number of data points required for the accurate representation of the figure.

### Questionnaires

#### Autism Spectrum Quotient (AQ)

The Autism Spectrum Quotient (AQ) is a self-report questionnaire designed to measure levels of autistic traits in adults of typical intelligent (IQ > 70) (Baron-Cohen et al., 2001). The questionnaire is composed of 50 questions, with five subscales with moderate internal consistency: social skills (Cronbach’s α = 0.77), attention-switching (Cronbach’s α = 0.67), attention to detail (Cronbach’s α = 0.63), communication (Cronbach’s α = 0.65) and imagination (Cronbach’s α = 0.65).

Questionnaires were scored using standard scoring systems (Baron-Cohen et al., 2001). Answers to each question of the AQ were scored as either 0 or 1 (see Appendix B for details). The maximum score is 50; however, scores over 26 are often considered to signify diagnosable autism (Woodbury-Smith et al., 2020). The AQ has been found to be a good tool for investigating the continuum of autistic expression in the general population and has been used extensively (Ruzich et al., 2016). Internal consistency and test-retest reliability of the questionnaire are largely consistent throughout different demographics and cultures (Broadbent et al., 2013; Lau et al., 2013; Stevenson & Hart, 2017).

#### Adult ADHD Self-Report Scale v1.1 (ASRS v1.1)

The Adult ADHD Self-Report Scale (Appendix B) is a self-report questionnaire consisting of 18 items with a Likert scale scoring system (Kessler et al., 2005). ASRS v1.1 has good sensitivity and specificity for detecting ADHD traits. Internal consistency ranges from 0.63 to 0.72 (Kessler et al., 2007). ASRS v1.1 (Kessler et al., 2005) is an 18-item questionnaire scoring between 0 and 4 (Appendix C). A maximum is 72; however, the first six questions are considered to be the most indicative of ADHD traits. This questionnaire has consistently shown high convergent validity, correlation of subscales and test-retest reliability throughout different demographics and cultures (Silverstein et al., 2017; Evren et al., 2016).

### Procedure

**Fig. 2:**
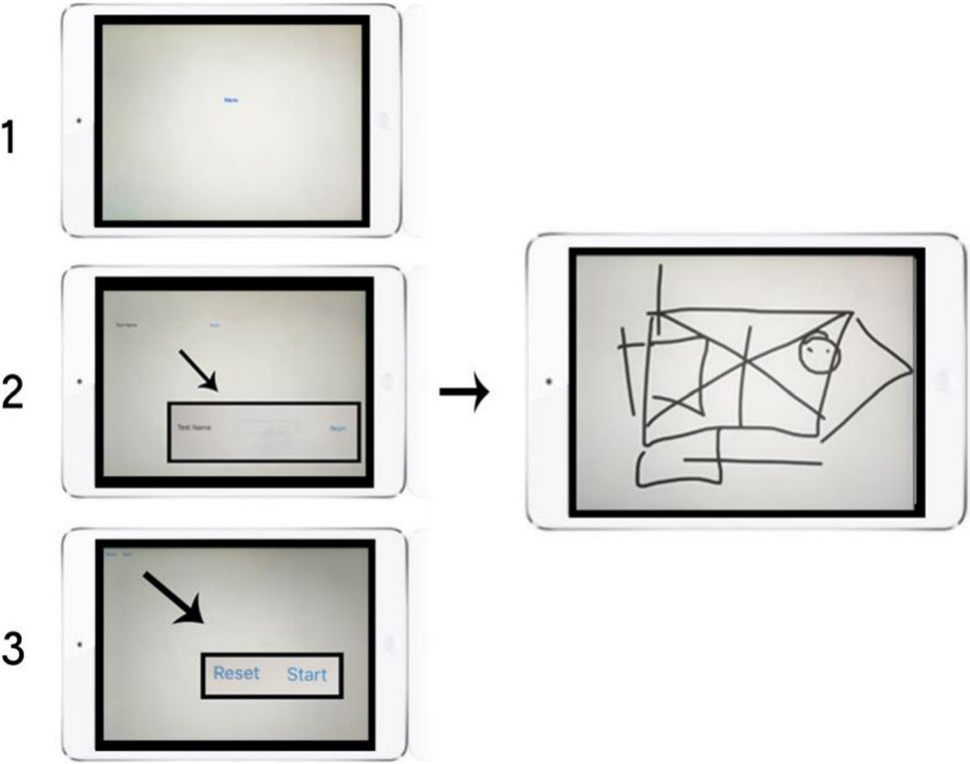
Stages of running the *LetsDraw* app: (1) new session started; (2) test name selected; (3) start and reset option available. The image on the right shows an example drawing from the feasibility study described later in the text

The study was conducted in accordance with the University of Glasgow and Economic and Social Research Council (ESRC) ethical guidelines. Participants gave informed consent before the experiment. The Rey-Osterrieth complex figure was presented on a computer screen and participants were asked to copy the figure using the *LetsDraw* app on an iPad mini (Fig. 2).

For the *Copy* condition, participants were seated in front of the computer, approximately 50 cm from the monitor. The ROCF image was presented as a full-screen image on a standard 23-inch LCD Acer monitor (resolution = 1920 × 1080 pixels, refresh rate = 59 Hz, mean luminance = 60 cd/m^2^), run on a Dell desktop computer. The ROCF image was a black line drawing on a white background. The iPad with the app already open and ready for drawing was placed in front of the participant.

After the *Copy* condition, the image on the computer screen was removed and participants were asked to draw the ROCF figure from memory. After the *Immediate Recall* condition, participants were asked to complete the AQ and ASRS questionnaires which were presented using Microsoft Forms on the computer. Once the questionnaires were complete, participants were asked to draw the ROCF figure from memory again. The delay time varied between participants, thus suggesting that the break was cognitive rather than temporal. This is a slight departure from the standardised procedure of the pen-and-paper ROCF task. Autistic and ADHD (Sørensen et al., 2017) individuals generally do not favour long delays between tasks; thus the decision was made to allow participants take breaks suitable for them. Once the *Delayed Recall* condition was completed, participants were debriefed. The whole experiment lasted approximately 30 minutes.

### ROCF scoring

**Fig. 3:**
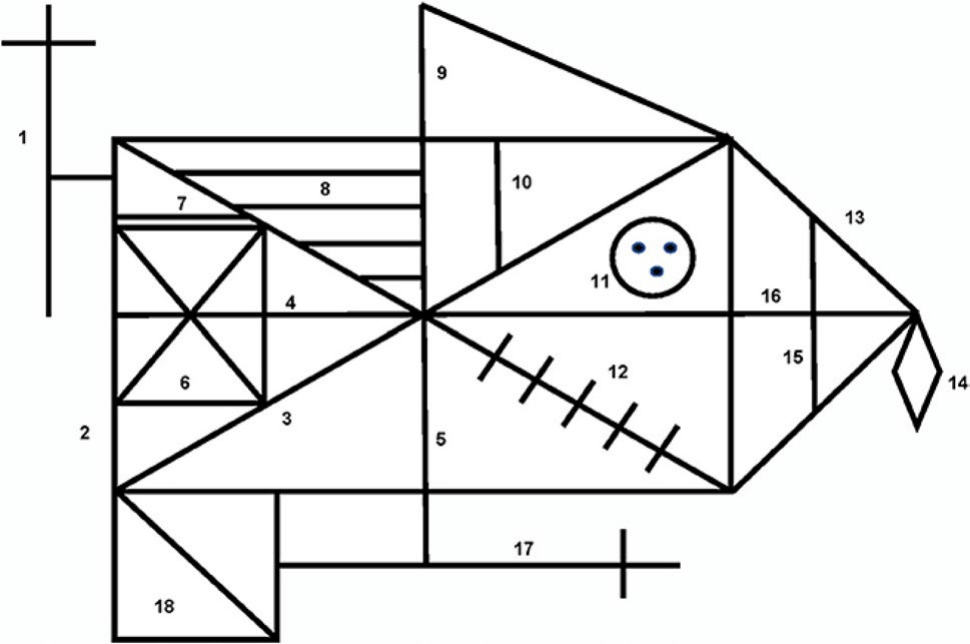
The perceptual scoring system for the ROCF (Booth, 2006; Osterrieth, 1944)

The perceptual scoring system was initially devised by Osterrieth (1944) and later adapted by Booth (2006). Each figure is scored using 18 features (Fig. 3). Two points are given if the feature is placed correctly, and one point given if it is incomplete or placed poorly. The maximum score possible is 36. The perceptual scoring system captures the accuracy of the task (Appendix C). This perceptual scoring system provides a quantitative measure of accuracy of reproduction of the task. It lacks qualitative analysis of performance and does not provide insight into other aspects of the task, such as processing style and planning subjectivities. However, inter-rater reliability can be as high as 0.99 (Mitrushina et al., 2005). Several qualitative scoring systems have been used alongside the standard perceptual scoring system (Osterrieth, 1944). Poreh and Ed.). (2012) describes numerous scoring systems with overlapping measures; however, they are all from the 1980s and have not re-emerged in recent literature.

An alternative is the Boston Qualitative Scoring system (BQS), which was devised by Stern et al. (1999), and it provides both quantitative summary scores and qualitative assessment of performance on the task. Inter-rater reliability determined by Kappa coefficients is high for most scores on the scale; however, some scores were considerably lower for scoring facets such as cluster and detail placement, rotation and neatness (Brauer Boone, 2000). Moreover, the BQS is not open-source and therefore could not be used in our study. As an alternative, we have selected the organisational scoring system by Hamby et al. (1993), which assesses the organisation and captures the quality of the approach taken to complete the task. It is often used alongside the BQS (Shin et al., 2006) and therefore, has been chosen as an alternative for our study.

The organisational scoring system was designed by Hamby et al. (1993) to evaluate organisational ability. Scores range from 1 to 5, where 1 is awarded for very poor organisation and 5 stands for excellent organisation. The full procedure for the organisational scoring is available in the online supplementary materials. The time element available from the *LetsDraw* app allows for more accurate organisational scoring, which relies on the order in which elements were drawn.

Two scorers, both authors on this paper, evaluated each drawing. No specialised training was required, as clear instructions on how scoring should be performed are available for both scoring systems (see online supplementary materials). Scorers assigned individuals scores independently and the mean score was used in further data analysis. There were no large discrepancies between the scores (Pearson’s correlation for differences in perceptual scores, *r*(115) = 0.96, *p* < 0.001; and Cohen’s kappa = 0.74, *p* < 0.001 for organisational scores). As the scoring systems used are quantitative in nature, the subjectivity of each scorer did not affect the overall result.

### Analytic plan

Shapiro–Wilk tests indicated that data were approximately normally distributed, allowing for the use of parametric inferential statistics. The independent variables (AQ and ASRS scores) were entered into multiple linear regression models to investigate how well they predicted the dependent variables (perceptual and organisational scores) across the experimental conditions.

## Results

**Fig. 4:**
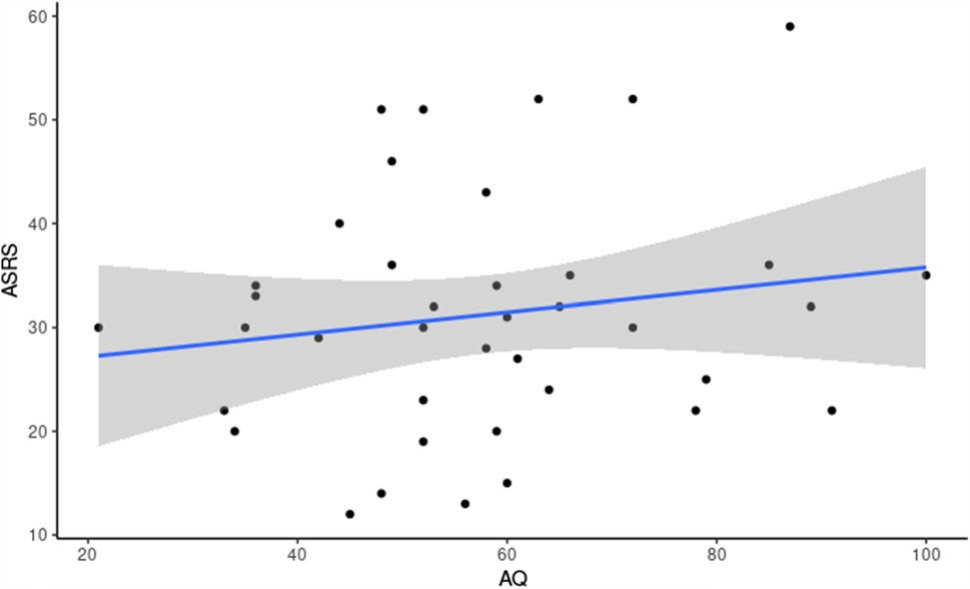
Scatterplot showing the relationships between AQ and ASRS questionnaire scores, *r*(37) = 0.22, *p* = 0.18

The internal consistency as measured using Cronbach’s alpha was good for both the AQ (α = 0.64) and ASRS questionnaires (α = 0.82). AQ scores ranged from 2 to 36, with a mean score for the sample of 16.82 (SD = 8.37). ASRS scores ranged from 12 to 59, with a mean score of 30.95 (SD = 11.59). Both AQ and ASRS scores demonstrated a wide range of traits in our sample. Scores of the two questionnaires did not show a significant correlation, *r*(37) = 0.22, *p* = 0.18 (Fig. 4).

**Fig. 5:**
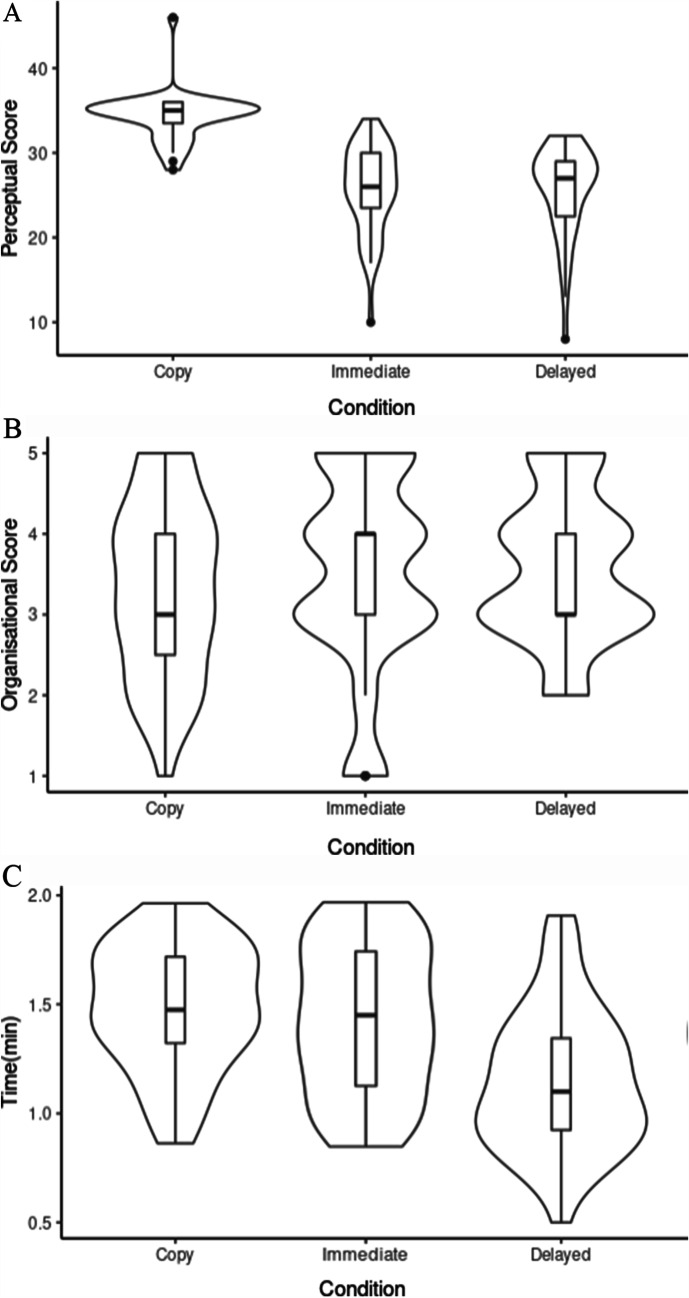
Visual comparison of ROCF scoring between *Copy*, *Immediate Recall* and *Delayed Recall* conditions. **a** Perceptual scores; **b** organisational scores, **c** completion time

The mean perceptual score for the *Copy* condition was 34.2 (SD = 1.93) and the mean score for organisational score was 3.33 (SD = 1.08). The *Copy* condition was completed on average in 89.2 s (SD = 17.1). A significant correlation was found between scores obtained using the two scoring systems, *r*(37) = 0.62, *p* = 0.001, for this condition. The mean perceptual score for the *Immediate Recall* condition was lower, 26.03 (SD = 0.504). However, the organisational score was slightly higher, 3.487 (SD = 1.2). Completion time was similar to the *Copy* condition with a mean time of 85.3 s (SD = 20.8). Organisational and perceptual scores correlated again, *r*(37) = 0.68, *p* = 2.136 × 10^−6^. The average perceptual score was lowest for the *Delayed Recall* condition, 25.9 (SD = 4.7). The organisational score was similar to the previous conditions, 3.56 (SD = 0.912). Completion time reduced to 68.6 s (SD = 19.1). A strong positive correlation was observed again between organisational and perceptual scores, *r*(37) = 0.61, *p* = 4.461e−5. Visual representation of the differences between the two scoring systems across all three experimental conditions is presented in Fig. 5.

To investigate the effects of the AQ and ADHD subscales on ROCF performance, two multiple linear regression models were implemented for perceptual and organisational scores, the results of which are presented in Table 1. The residuals of the models were normally distributed. The models were found to be significant for both perceptual (adjusted *R*^2^ = 0.47, *F*(9, 107) = 12.48, *p* < 0.001) and organisational score (adjusted *R*^2^ = 0.19, *F*(9, 107) = 4.10, *p* < 0.001). Significance for the model of perceptual scores seems to have been mainly driven by the differences in perceptual scores between the conditions. Table 1A shows significant differences between the *Copy* condition and both *Immediate Recall* and *Delayed Recall* conditions. This is further illustrated by Fig. 5a, where this difference can be visualised. Organisational scores, however, did not show a significant difference between the three experimental conditions (Fig. 5b and Table 1B). The significance of the model seems to have been driven by AQ subscales, where attention to detail (higher score = higher attention to detail) and communication (higher score = difficulties with communication) were positive predictors and attention-switching (higher score = difficulties with attention-switching) was a negative predictor of the performance on the task. Higher scores on attention to detail and communication have successfully predicted higher organisational scores, suggesting that this subscale is important in global processing tasks, whereas higher scores on the attention-switching subscale predicted lower organisational scores and therefore local processing bias Table 2.

**Table 1:**
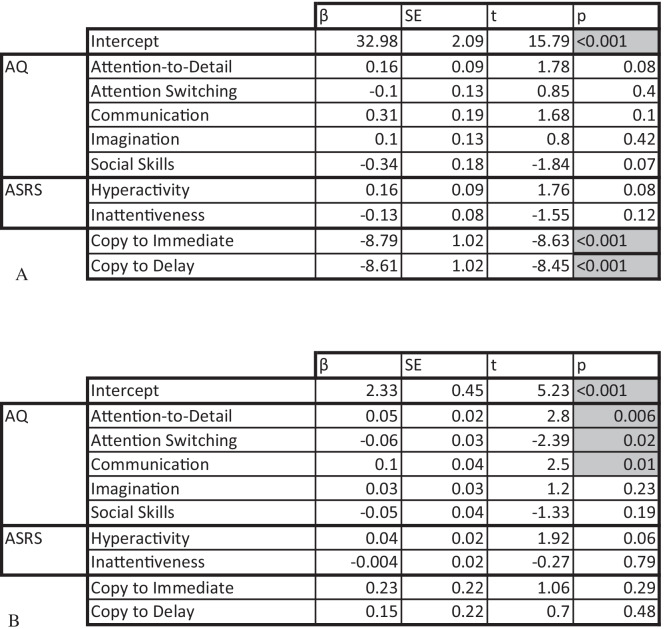
Coefficients from multiple linear regressions of perceptual (A) and organisational (B) ROCF scores. Significant predictors are highlighted.

**Fig. 6:**
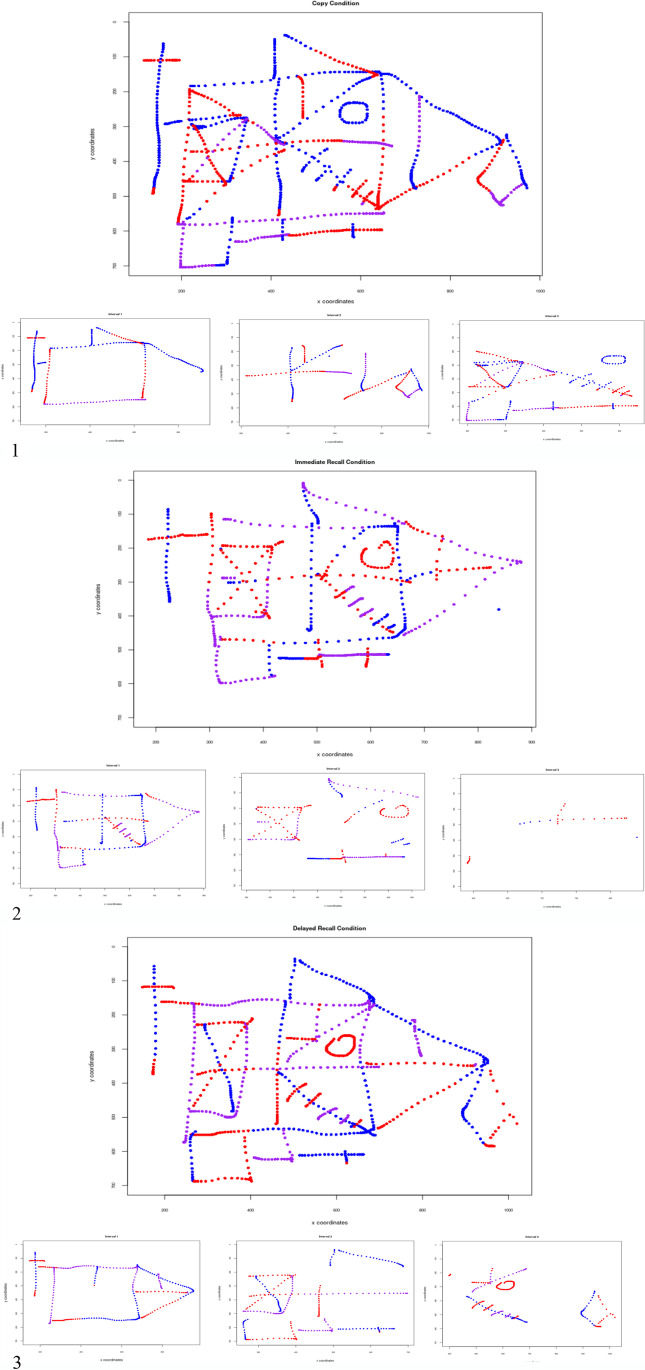
Visual recreation of drawings made by Participant 3 for all experimental conditions: *Copy* (1), *Immediate Recall* (2) and *Delayed Recall* (3). Additional subplots represent elements drawn at separate time intervals. The colour transitions represent how the figure changed over time (blue – elements drawn first, red – elements drawn last).

Coordinates were plotted with the time element to visualise the data. An example of the visualisation is presented in Table 1. Introducing completion time allows precise visualisation of the way participants completed the drawing for each individual condition. In Fig. 6 we present data from Participant 3 and break down the overall performance into three equal time intervals to identify which elements of the ROCF were drawn first. The *Copy* and *Immediate Recall* conditions appear to show some global preference, where larger elements, such as the rectangle and longer definitive lines, are drawn first. This preference appears to be more distinct in the *Delayed Recall* condition. A tendency towards a more global processing style in the *Delayed* condition is reflected in the organisational scores (see Fig. 5b).

**Fig. 7:**
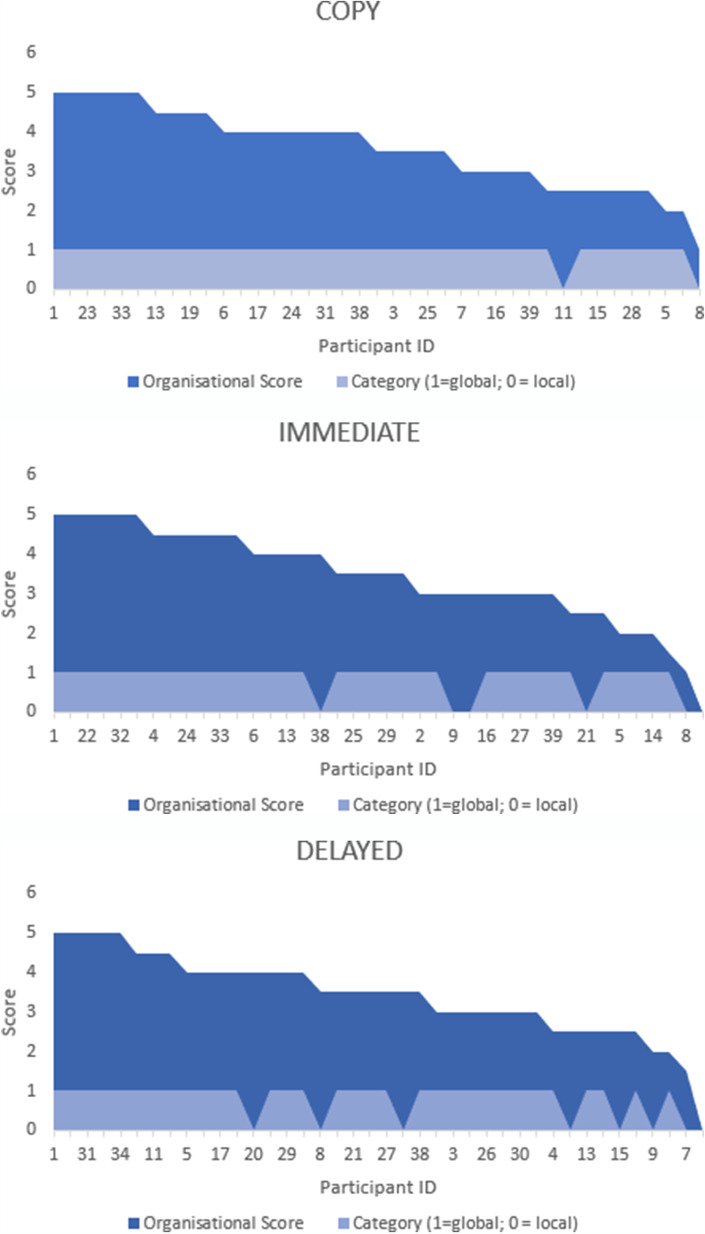
Graphical representation of the relationship between organisational score and the assigned category (1 = global processing style; 0 = local processing style) to individual drawings of each participant by condition. Organisational scores have been arranged in descending order to illustrate the relationship between processing style and the score.

In order to assess how the temporal data compared to the organisational scores, all of the drawings were coded in a binary code, where a score of 1 (global processing) was assigned to drawings where global elements were drawn first, and a score of 0 (local processing) was assigned to drawings where local elements were drawn first. These were plotted against organisational scores (Fig. 7).

## Discussion

The aim of this feasibility study was to explore the utility of an iPad-based ROCF task. We were also interested in the relationship between autistic/ADHD traits and ROCF scores obtained with this new digital method.

In terms of feasibility, participants were happy to use the *LetsDraw* app to perform the task. The key advantage of the app over traditional colour-coded crayon-based methods (e.g. Tsatsanis et al., 2011), is that temporal aspects of the drawing sequence can be reconstructed in full, without having to video-record the participant, as shown in Fig. 6, and this advantage was realised in our data analysis. Figure 7 is a graphical representation of the relationship between organisational score and the assigned category (1 = global processing style; 0 = local processing style) to individual drawings of each participant by condition. Organisational scores have been arranged in descending order to illustrate the relationship between processing style and score. Organisational scores had fairly similar distributions throughout the three experimental conditions, although only *Immediate Recall*–*Copy* scores correlated significantly, *r*(37) = 0.75, *p* < 0.01. It is evident, however, that the number of drawings assigned to the local processing category increase in the *Delayed Recall* condition, suggesting that participants seem to adopt a different strategy for drawing from memory.

As for the results we obtained, although diagnosed autism and ADHD have previously been found to be related conditions (Chantiluke et al., 2014), there was no significant correlation between AQ and ASRS scores in our sample. We have used a sample with variable autistic and ADHD traits. Although a relationship between autism and ADHD has been previously observed in the general population (Geurts et al., 2013), it is possible that autistic and ADHD traits correlate in domains not captured by the questionnaires we used, or there could be additional confounding variables contributing to the results we have observed.

ROCF *perceptual scores* and *completion time* were significantly different between the conditions. In contrast, ROCF *organisational scores* did not show this pattern (see Table 1). These results, with the exception of organisational score, were expected at the start of the experiment. The *Copy* condition often takes the longest to process as it is an abstract figure that most participants have never seen before. It is also the most accurate as the image is visible and participants do not have to rely on their memory.

**Fig. 8:**
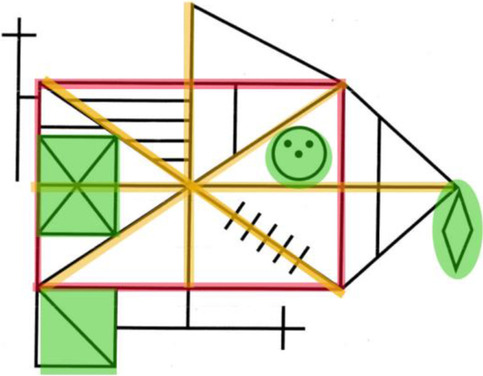
Visual representation of global processing style of the ROCF. Red and yellow lines indicate elements drawn first and green highlights details completed last.

AQ and ASRS subscales did not predict perceptual ROCF scores, however, an interesting pattern emerged for the organisational ROCF scores. The attention-to-detail, attention-switching and communication subscales of the AQ were found to be predictive of organisational ROCF scores. The organisational scoring system (see online supplementary materials for full details) awards higher scores for a global completion of ROCF task. In other words, if the participant draws the rectangle and cross-over lines first and fills in smaller details (“little face”, diamond and small boxes) later, the maximum points are awarded (Fig. 8). Points can be reduced if lines do not meet and smaller elements are disconnected. In our data, if participants scored higher on the attention-to-detail AQ subscale their organisational scores were significantly higher. Although previous findings have identified differences between the attention-to-detail subscale of the AQ in facial recognition (Davis et al., 2017), our study demonstrates a new idea that this subscale can be linked to visual processing and individual cognitive styles in a general population sample (see also Van Eylen et al., 2018).

Moreover, the attention-switching subscale of the AQ had a significant negative relationship with the organisational scores. High scores on this subscale of the AQ suggest poorer attention-switching ability. Our finding demonstrates that participants with flexible attention-switching had lower organisational scores, potentially suggesting that they have adopted a local processing style. The attention-switching subscale has been previously linked to ADHD (Cepeda, Cepeda & Kramer, 2000; Dibbets et al., 2010), however, our findings do not support this link (as the AQ and ASRS questionnaires did not show a significant correlation). Perhaps, previous links between AQ and ASRS questionnaires have been driven by the subscales of the AQ. Previous work suggests that ADHD and autism might share common aetiology; however, it is not clear how each subscale of the two questionnaires are related (Concerto et al., 2021; Dalbudak & Evren, 2014; Panagiotidi et al., 2018; Panagiotidi et al., 2019).

Finally, the communication subscale was also found to be a significant predictor of participants’ performance and organisational scores. Higher scores on the communication subscale of the AQ suggest greater challenges with communication. Participants with higher scores on the communication subscale were more organised and had higher organisational scores in our sample. Challenges with social cognition, emotion recognition and communication skills have been previously linked to autistic traits, but results are inconclusive (McKenzie et al., 2018; Oerlemans et al., 2013).

Organisational scores were expected to correlate negatively with the AQ (Luna et al., 2007); however, our results suggest that higher autistic trait levels were associated with better organisation. Moreover, completion times were no longer in participants with higher AQ scores, contradicting our prediction that autistic traits affect executive function and specifically motivation in the task (Ferraro et al., 2018). Our results challenge the notion that autistic traits are associated with reduced global and enhanced local processing as proposed by the Weak Coherence theory that attends exclusively to perceptual information (Shah & Frith, 1993). Similar results for a different task have been found by Hayward et al. (2018).

## Limitations

One of the limitations of the current study is its relatively small sample size. A group of 39 participants does not meet the recommended number for correlations (Bonett & Wright, 2000) or multiple regression models (VanVoorhis & Morgan, 2007). However, previous ROCF studies have successfully used similar sample sizes (*N* = 37, see Kuschner et al., 2009). Moreover, our sample solely consisted of undergraduate students, and thus our results could not be generalised to the general population. Participants were mostly female and the age range of 18–25 was limited. However, as noted previously, ADHD and autism are both neurodevelopmental conditions and therefore the presentation may follow different developmental trajectories for males and females, with earlier onset for males. Given that the ROCF will likely be of interest to child/adolescent providers, these developmental considerations will need to be accounted for when using the ROCF app with these younger populations. We did not collect additional information on participants’ mental health. Many other conditions have been previously linked to variable performance in ROCF task, such as eating disorders (Eisenberg et al., 2011; Lang et al., 2016).

The technology employed in similar, recent drawing experiments is mixed. There is no consistency in medium: some use older models of touch-screen technologies, others incorporate a pen or a stylus into their studies (Hyun et al., 2018). Future studies should explore the advantages and disadvantages between media in more depth, especially as drawing with a finger on a touch screen involves different muscle groups and different friction characteristics from drawing with a pen. Moreover, the tactile response from traditional pen and paper drawing will differ from the pen/stylus used in many of the tablets used today (Kirkorian et al., 2020)

Finally, the surprising findings of the link between scores on the attention-to-detail subscale of the AQ and organisational scores warrant further investigation. Questionnaires with focus on executive function, such as the Executive Function Index (EFI; Ferraro et al., 2018), should be employed in order to further explore the meaning of this effect.

## Conclusion

The *LetsDraw* app is a novel data collection tool which enables the fast visualisation and analysis of drawing tasks. This feasibility study has highlighted probable associations between higher autistic traits and organisational performance in the ROCF task. An association between autistic traits and time to complete the task was, however, not supported. ADHD traits were not found to be associated with perceptual and organisational scores in the task, nor the time it took to complete the task. These results provide a preliminary suggestion that autistic traits are in some way related to enhanced abilities in perceiving local and global aspects of the figure and relate higher autistic trait levels to better organisation, contrary to some existing theories of autistic perception.

Further feasibility studies of the new methodology used in this experiment should be explored. Data extracted from this digital translation of the ROCF lends itself to further, additional analyses. New computational measures include accuracy and computation of the fine motor control kinematics employed to carry out the drawing, include the possibility to include and test theories of the prospective organisation of movement thought to be disrupted in autism, but not in ADHD (Trevarthen & Delafield-Butt, 2013). The addition of the Apple Pencil would afford pressure detection, which is important in motor organisation. Ultimately, the task’s metrics of interest may be automated to allow quicker identification of visual processing strategies adopted. It can also be further adapted to explore alternative drawing tasks to shed light on perceptuo-motor properties of neurodevelopmental conditions.

## Appendix A

**Table 2:** AQ Questionnaire

		definitely agree	slightly agree	slightly disagree	definitely disagree
1.	I prefer to do things with others rather than on my own.			1	1
2.	I prefer to do things the same way over and over again.	1	1		
3.	If I try to imagine something, I find it very easy to create a picture in my mind.			1	1
4.	I frequently get so strongly absorbed in one thing that I lose sight of other things.	1	1		
5.	I often notice small sounds when others do not.	1	1		
6.	I usually notice car number plates or similar strings of information.	1	1		
7.	Other people frequently tell me that what I’ve said is impolite, even though I think it is polite.	1	1		
8.	When I’m reading a story, I can easily imagine what the characters might look like.			1	1
9.	I am fascinated by dates.	1	1		
10.	In a social group, I can easily keep track of several different people’s conversations.			1	1
11.	I find social situations easy.			1	1
12.	I tend to notice details that others do not.	1	1		
13.	I would rather go to a library than a party.	1	1		
14.	I find making up stories easy.			1	1
15.	I find myself drawn more strongly to people than to things.			1	1
16.	I tend to have very strong interests which I get upset about if I can’t pursue.	1	1		
17.	I enjoy social chit-chat.			1	1
18.	When I talk, it isn’t always easy for others to get a word in edgeways.	1	1		
19.	I am fascinated by numbers.	1	1		
20.	When I’m reading a story, I find it difficult to work out the characters’ intentions.	1	1		
21.	I don’t particularly enjoy reading fiction.	1	1		
22.	I find it hard to make new friends.	1	1		
23.	I notice patterns in things all the time.	1	1		
24.	I would rather go to the theatre than a museum.			1	1
25.	It does not upset me if my daily routine is disturbed.			1	1
26.	I frequently find that I don’t know how to keep a conversation going.	1	1		
27.	I find it easy to “read between the lines” when someone is talking to me.			1	1
28.	I usually concentrate more on the whole picture, rather than the small details.			1	1
29.	I am not very good at remembering phone numbers.			1	1
30.	I don’t usually notice small changes in a situation, or a person’s appearance.			1	1
31.	I know how to tell if someone listening to me is getting bored.			1	1
32.	I find it easy to do more than one thing at once.			1	1
33.	When I talk on the phone, I’m not sure when it’s my turn to speak.	1	1		
34.	I enjoy doing things spontaneously.			1	1
35.	I am often the last to understand the point of a joke.	1	1		
36.	I find it easy to work out what someone is thinking or feeling just by looking at their face.			1	1
37.	If there is an interruption, I can switch back to what I was doing very quickly.			1	1
38.	I am good at social chit-chat.			1	1
39.	People often tell me that I keep going on and on about the same thing.	1	1		
40.	When I was young, I used to enjoy playing games involving pretending with other children.			1	1
41.	I like to collect information about categories of things (e.g. types of car, types of bird, types of train, types of plant, etc.).	1	1		
42.	I find it difficult to imagine what it would be like to be someone else.	1	1		
43.	I like to plan any activities I participate in carefully.	1	1		
44.	I enjoy social occasions.			1	1
45.	I find it difficult to work out people’s intentions.	1	1		
46.	New situations make me anxious.	1	1		
47.	I enjoy meeting new people.			1	1
48.	I am a good diplomat.			1	1
49.	I am not very good at remembering people’s date of birth.			1	1
50.	I find it very easy to play games with children that involve pretending.			1	1

## Appendix C – ROCF Organizational Scoring

(Hamby et al., 1993)

There are three types of mistakes: configural, secondary, and detail mistakes, defined below. A person's score depends on the number and type of mistakes in the drawing, as follows:

First, *count the number of configural mistakes.*

- If there are three or more configural mistakes, score 1 for very poor organization. Stop.

1. If there are 0, 1, or 2 configural mistakes, *check for a secondary mistake. If present, add to the number of configural mistakes.*
  - If the total is 3, then score 1 for very poor organization. Stop.
  - If the total is 2, then score 2 for poor organization. Stop.
  - If the total is 1, then score 3 for fair organization. Stop
2. If there are 0 configural and secondary mistakes, *check for detail mistakes.*
  - If there are 1 or more detail mistakes, score 4 for good organization. Stop.
  - If there are 0 detail mistakes, score 5 for excellent organization. Stop.

**Configural Mistakes**

The basis configural elements of the Rey-Osterrieth are the outer rectangle and the vertical and horizontal midlines.

1. The following errors are considered configural mistakes (no line can count for more than 1 mistake):

- one side of rectangle/square not drawn when others are completed.
- either of the following drawn in two or more segments constitutes 1 mistake (maximum = 6 mistakes): four sides of rectangle/square or two midlines (i.e., it should only take six lines to complete all of the elements above).
- any details completed before the configural elements are completed, *except for the upper left cross (No. 1) of the Rey-Osterrieth.* (Because starting in the upper left-hand corner seems to be more related to standard Western writing practice than to poor organization, it is not penalized as heavily.) For example, drawing the right triangle (No. 13) before completing the vertical midline would constitute a configural mistake. Count *one* mistake for each configural line drawn after the details have been drawn. For example, if the right triangle (No. 13) of the Rey-Osterrieth is begun before *both* midlines are drawn, score as 2 configural mistakes.
- either midline drawn more than 10% away from the center (in either direction). That is, the midlines should be in the central 40–60% of the rectangle/square.
- sides of the rectangle/square not joined at their endpoints, suggesting that the subject does not perceive the rectangle/square. Small draftsmanship errors should not count as a configural mistake. For example, if the right side is placed too far to the right, creating a 6-sided figure, that would count as one configural mistake. Each poorly placed line counts as 1 mistake.
- a configural element is missing (score 1).

**Secondary Mistakes**

The diagonals (No. 3) of the Rey-Osterrieth are considered to be secondary elements.

1. Two errors in the reproduction of the secondary elements are considered serious enough to be counted as a secondary mistake, which carries the same weight as configural mistakes. However, the overall construction of these elements can count as a maximum of one mistake. The mistakes are the following:
  - the segments do not meet. For the Rey-Osterrieth, all 4 slashes of the diagonal should meet.
  - a segment is incomplete (i.e., does not extend all the way across figure).
2. When the elements are drawn as connecting segments, score as a detail mistake. For the Rey-Osterrieth, score as a detail mistake if the patient uses more than two lines to complete the diagonals.
3. Secondary elements can be completed before midlines with no penalty.

**Detail Mistakes**

All other elements are considered to be details.

1. Detail mistakes can be made in three ways:
  - Unnecessary segmentation. The participant should not use more lines than necessary to complete the element.
  - Lines in a standard element, and elements that are near each other, should be drawn consecutively.
  - Poor planning that results in the need to redraw an element. For instance, if they must redraw a diagonal line so that it intersects both corners.
2. The following is a partial list of common detail mistakes found in reproductions of the Rey-Osterrieth No. 1 Cross completed before configural elements .No. 6

- 3 sides of box are not done together (with no break at midline)
- X is not completed immediately after box is drawn
- X is drawn as four slashes or two V's (rather than two intersecting lines).

No. 7 Not done immediately after No. 6.

No. 9 Both sides of triangle not completed together. Vertical side drawn as part of midline and then slanted side completed later.

Nos. 9, 13 The adjacent sides of these two triangles drawn as a single line.

No. 13 Both sides of this triangle not completed together.

Nos. 13, 15, 16 Not drawn together.

No. 17 Attachment for cross not completed with rest of cross (e.g., done as part of midline and then finished later).

No. 18

- Slash in box not added when box is first drawn.
- Box, or part of box, drawn as part of the basic configural rectangle.

## Appendix D – ROCF Perceptual Scoring

(Adapted from Osterrieth, 1944)

## Footnotes

1 ASD, autism and individuals with autism will be used interchangeably in this paper. Recent research on neurodiversity and person-first vs identity-first language suggests this is an appropriate use of terminology (see Bury, Jellett, Spoor & Hedley, 2020).

2 XCode is an integrated development environment developed for macOS by Apple in 2003. It predominantly uses the Swift programming language, which is based on C++ and Java.

3 *Home* device (i.e. laptop or desktop computer) and *test* device (i.e. iPad or iPhone) will be used as shorthand terms in this paper to describe the device containing the XCode (*home*) and the device which is used to run the experiment (*test*).

4 .csv file format stands for comma-separated values, and it is a delimited text file which uses commas to separate values. It is the file format used for the majority of data analysis programmes, including R.
